# Reducing Aspergillus fumigatus Virulence through Targeted Dysregulation of the Conidiation Pathway

**DOI:** 10.1128/mBio.03202-19

**Published:** 2020-02-04

**Authors:** James I. P. Stewart, Vinicius M. Fava, Joshua D. Kerkaert, Adithya S. Subramanian, Fabrice N. Gravelat, Melanie Lehoux, P. Lynne Howell, Robert A. Cramer, Donald C. Sheppard

**Affiliations:** a Department of Experimental Medicine, McGill University, Glen Site, Research Institute of the McGill University Health Centre, Montreal, Quebec, Canada; b Infectious Diseases and Immunity in Global Health Program, Glen Site, Research Institute of the McGill University Health Centre, Montreal, Quebec, Canada; c Departments of Medicine and of Microbiology and Immunology, McGill University, Glen Site, Research Institute of the McGill University Health Centre, Montreal, Quebec, Canada; d McGill Interdisciplinary Initiative in Infection and Immunity, Montreal, Quebec, Canada; e McGill International TB Centre, Glen Site, Research Institute of the McGill University Health Centre, Montreal, Quebec, Canada; f Department of Microbiology and Immunology, Geisel School of Medicine at Dartmouth, Hanover, New Hampshire, USA; g Program in Molecular Medicine, Research Institute, The Hospital for Sick Children, Toronto, Ontario, Canada; h Department of Biochemistry, Faculty of Medicine, University of Toronto, Toronto, Ontario, Canada; University of British Columbia

**Keywords:** *Aspergillus fumigatus*, RNA sequencing, conidiation, drug targets, inducible gene expression, opportunistic pathogen, virulence

## Abstract

Inhalation of conidia of the opportunistic mold Aspergillus fumigatus by immunocompromised hosts can lead to invasive pulmonary disease. Inhaled conidia that escape immune defenses germinate to form filamentous hyphae that invade lung tissues. Conidiation rarely occurs during invasive infection of the human host, allowing the bulk of fungal energy to be directed toward vegetative growth. We hypothesized that forced induction of conidiation during infection can suppress A. fumigatus vegetative growth, impairing the ability of this organism to cause disease. To study the effects of conidiation pathway dysregulation on A. fumigatus virulence, a key transcriptional regulator of conidiation (*brlA*) was expressed under the control of a doxycycline-inducible promoter. Time- and dose-dependent *brlA* overexpression was observed in response to doxycycline both *in vitro* and *in vivo.* Exposure of the inducible *brlA* overexpression strain to low doses of doxycycline under vegetative growth conditions *in vitro* induced conidiation, whereas high doses arrested growth. Overexpression of *brlA* attenuated A. fumigatus virulence in both an invertebrate and mouse model of invasive aspergillosis. RNA sequencing studies and phenotypic analysis revealed that *brlA* overexpression results in altered cell signaling, amino acid, and carbohydrate metabolism, including a marked upregulation of trehalose biosynthesis and a downregulation in the biosynthesis of the polysaccharide virulence factor galactosaminogalactan. This proof of concept study demonstrates that activation of the conidiation pathway in A. fumigatus can reduce virulence and suggests that *brlA*-inducing small molecules may hold promise as a new class of therapeutics for A. fumigatus infection.

## Abstract (precis)

The mold Aspergillus fumigatus reproduces by the production of airborne spores (conidia), a process termed conidiation. In immunocompromised individuals, inhaled A. fumigatus conidia can germinate and form filaments that penetrate and damage lung tissues; however, conidiation does not occur during invasive infection. In this study, we demonstrate that forced activation of conidiation in filaments of A. fumigatus can arrest their growth and impair the ability of this fungus to cause disease in both an insect and a mouse model of invasive infection. Activation of conidiation was linked to profound changes in A. fumigatus metabolism, including a shift away from the synthesis of polysaccharides required for cell wall structure and virulence in favor of carbohydrates used for energy storage and stress resistance. Collectively, these findings suggest that activation of the conidiation pathway may be a promising approach for the development of new agents to prevent or treat A. fumigatus infection.

## INTRODUCTION

Aspergillus fumigatus is a ubiquitous mold that reproduces by producing airborne conidia (1). While inhaled A. fumigatus conidia are rapidly cleared via innate immune defenses in healthy individuals (2–5), in immunocompromised patients conidia can germinate to form filamentous hyphae that invade lung tissues, causing a necrotizing pneumonia that is associated with high mortality rates (6–9).

Hyphae are the predominant fungal morphology observed during invasive pulmonary aspergillosis, while conidiation is rarely observed (10). During growth *in vitro*, A. fumigatus conidiation is associated with a marked reduction in hyphal growth (11). We therefore hypothesized that the forced induction of the conidiation pathway during infection could also suppress hyphal growth and thereby attenuate A. fumigatus virulence. Control of conidiation in *Aspergillus* spp. has been well studied in the model organism Aspergillus nidulans where the transcription factor BrlA has been identified as a master regulator of conidiation. BrlA-deficient A. nidulans does not conidiate, whereas *brlA* overexpression rapidly induces conidiation and inhibits the growth of hyphae *in vitro* (12). An A. fumigatus Δ*brlA* mutant also does not conidiate, has increased hyphal mass (11, 13), and exhibits widespread transcriptional dysregulation of genes linked to conidiation, growth, and virulence (14). However, the effects of *brlA* overexpression in A. fumigatus are unknown.

In this study, we demonstrate that targeted upregulation of *brlA* is sufficient to induce conidiation and inhibit A. fumigatus growth *in vitro*. Overexpression of *brlA* significantly reduces the virulence of A. fumigatus in an invertebrate and mouse model of invasive aspergillosis. RNA sequencing (RNA-seq) analysis demonstrated that overexpression of *brlA* resulted in significant changes in the expression of genes involved in cell signaling, carbon, and nitrogen metabolism, including a shift in carbohydrate metabolism away from cell wall polysaccharide synthesis and toward the production of storage carbohydrates. Phenotypic analyses confirmed that *brlA* overexpression increases hyphal trehalose content and reduces levels of cell wall galactosaminogalactan, possibly contributing to *brlA*-dependent attenuation of virulence.

## RESULTS

### Doxycycline-mediated induction of *brlA* in *A. fumigatus* is both time and dose dependent.

**FIG 1:**
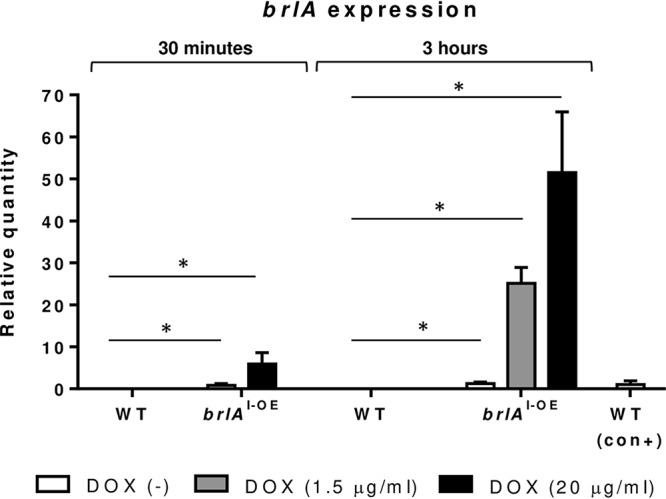
Construction of a doxycycline-inducible *brlA* overexpression mutant of A. fumigatus. The expression of *brlA* in parental wild-type Af293 (WT), inducible *brlA*-overexpressing (*brlA*^I-OE^), and conidiating wild-type Af293 (WT con+) strains of A. fumigatus after 30 min or 3 h of exposure to the indicated concentrations of doxycycline was measured by RT-qPCR. Gene expression was normalized to the endogenous reference gene *tef1* and is presented as the fold change relative to the conidiating wild-type strain. The combined data for three independent experiments are presented as the means and standard errors of three biological replicates, each performed in triplicate. *, *P < *0.0001 (two-tailed Student *t* test). *brlA*^I-OE^ DOX (–), *brlA*^I-OE^ DOX (1.5 μg/ml), and *brlA*^I-OE^ DOX (20 μg/ml) were compared to wild-type DOX (–) at the same time point.

To investigate the effects of *brlA* overexpression on the growth and conidiation of A. fumigatus, an inducible *brlA* overexpression (*brlA*^I-OE^) strain was constructed in which *brlA* was placed under the control of a doxycycline-inducible promoter. PCR screening of genomic DNA (gDNA) from the *brlA*^I-OE^ mutant confirmed the presence of the linear Tet-ON-*brlA* system, as well as the absence of a circular autonomously replicating Tet-ON-*brlA* plasmid. The presence of the native *brlA* open reading frame (ORF) was also confirmed in the *brlA*^I-OE^ mutant, suggesting ectopic integration of the Tet-ON-*brlA* system. Doxycycline treatment of the *brlA*^I-OE^ mutant resulted in dose- and time-dependent overexpression of *brlA* in this strain (Fig. 1). As previously reported with this system (15), increased basal *brlA* expression was also observed in this strain in the absence of doxycycline. This strain was used for further analyses of the effects of *brlA* overexpression on A. fumigatus fitness *in vitro* and *in vivo*.

### Overexpression of *brlA* inhibits the growth of precompetent *A. fumigatus* hyphae *in vitro*.

**FIG 2:**
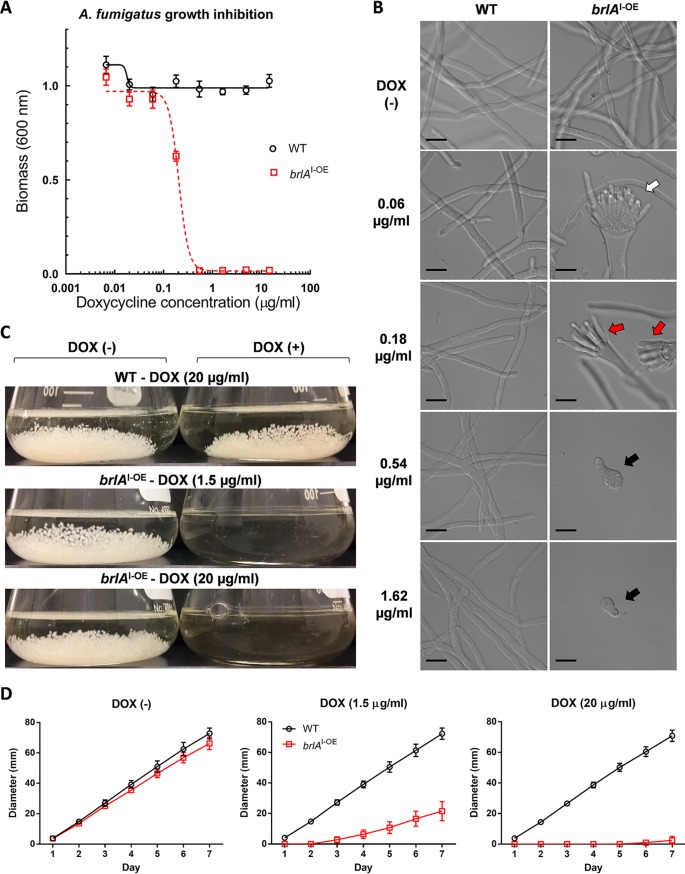
Overexpression of *brlA* in precompetent A. fumigatus hyphae results in dose-dependent growth inhibition. The effects of *brlA* overexpression on the growth and conidiation of precompetent A. fumigatus hyphae *in vitro* were assessed. (A) Crystal violet staining of the biomass of the parental wild-type Af293 (WT) and inducible *brlA*-overexpressing (*brlA*^I-OE^) strains after 20 h of exposure to the indicated concentrations of doxycycline. Each data point represents the mean and standard error of 3 biological replicates each with four to five technical replicates. (B) Differential interference contrast imaging of the WT and *brlA*^I-OE^ strains after 20 h of exposure to the indicated concentrations of doxycycline. White arrows indicate normal conidiophore formation, red arrows indicate stunted atypical conidiophore formation, and black arrows indicate hyphae that have undergone growth arrest. Scale bars, 10 μm. (C) Liquid shaking cultures of the WT and *brlA*^I-OE^ strains after 24 h of exposure to the indicated concentrations of doxycycline. (D) Radial growth rate of the WT and *brlA*^I-OE^ strains over 7 days of exposure to the indicated concentrations of doxycycline on solid AMM media. Each data point represents the mean and standard deviation of four biological replicates.

The development of A. fumigatus hyphae begins with a genetically defined precompetent period, during which hyphae are unable to conidiate in response to stimuli (16–20). To determine the effect of *brlA* overexpression on the growth of precompetent A. fumigatus, conidia were exposed to a range of doxycycline concentrations in static culture, and the resulting biomass was quantified by crystal violet staining (Fig. 2A) and microscopy (Fig. 2B). Under these conditions, wild-type A. fumigatus and the uninduced *brlA*^I-OE^ mutant underwent normal germination and hyphal growth without conidiation (Fig. 2A and B). In contrast, the *brlA*^I-OE^ mutant exhibited doxycycline dose-dependent growth inhibition, with an MIC of 0.54 μg/ml doxycycline (Fig. 2A). Microscopy revealed that at doxycycline concentrations of 0.54 μg/ml and above, *brlA*^I-OE^ conidia underwent germination but exhibited arrested hyphal growth (Fig. 2B). At lower doxycycline concentrations, *brlA*^I-OE^ cultures underwent conidiation, although at 0.18 μg/ml of doxycycline *brlA*^I-OE^ conidiophore morphology was atypical, with reduced vesicle size and elongated phialides (Fig. 2B). Thus, lower levels of *brlA* induction in precompetent A. fumigatus hyphae reduces vegetative growth and induces conidiation *in vitro*, whereas higher levels result in the complete inhibition of vegetative growth.

Analysis of the effects of *brlA* overexpression on A. fumigatus growth and conidiation under liquid static conditions were limited to 24 h due to conidiation of the wild-type strain. To assess the effects of *brlA* overexpression at later time points, A. fumigatus conidia were grown in liquid shaking culture with or without doxycycline for 48 h (Fig. 2C and see Fig. S1A in the supplemental material). Under these conditions, doxycycline inhibited growth of the *brlA*^I-OE^ strain for 24 h (Fig. 2C). At 48 h of growth, however, breakthrough clusters of *brlA*^I-OE^ hyphae were observed. These hyphal masses exhibited reduced growth and increased conidiation compared to wild-type hyphae that was more apparent in the presence of the higher concentration of doxycycline (Fig. S1A). These findings suggest that the level of *brlA* overexpression may influence the duration of growth inhibition, as well as the rate of vegetative growth and induction of conidiation thereafter.

10.1128/mBio.03202-19.1 FIG S1 Precompetent A. fumigatus hyphal breakthrough of *brlA* overexpression-induced growth inhibition is dose dependent. (A) Effects of *brlA* overexpression on the growth and conidiation of precompetent A. fumigatus in liquid culture. Visual inspection of liquid shaking cultures of the parental wild-type Af293 (WT) and the inducible *brlA* overexpressing (*brlA*^I-OE^) strains after exposure to indicated concentrations of doxycycline for 48 h. (B) Radial growth rate of the WT and *brlA*^I-OE^ strains over 7 days of exposure to the indicated concentrations of doxycycline on solid YPD media. Each data point represents the mean and standard deviation of four biological replicates. (C) Response to doxycycline as expression of *brlA* as measured by RT-qPCR after exposure of the indicated strains of A. fumigatus to 20 μg/ml doxycycline for 3 h. Strains tested include vegetative hyphae of the parental wild-type Af293 (WT) and inducible *brlA*-overexpressing (*brlA*^I-OE^) strains, conidiating wild-type Af293 (WT con+) and five breakthrough mutants (*brlA*^BT^ 1, 2, 3, 4, and 5) isolated from pre-grown competent *brlA*^I-OE^ hyphae. Gene expression was normalized to the endogenous reference gene *tef1* and presented as fold change relative to the conidiating wild-type strain. The combined data of three independent experiments are presented as the means and the standard errors of the delta *C_T_* of three biological replicates, each performed in triplicate. *, *P < *0.001; **, *P < *0.0001 (two-tailed Student t-test comparing each strain treated with doxycycline to doxycycline-free controls at the same time point). Download FIG S1, TIF file, 0.2 MB. Copyright © 2020 Stewart et al. 2020 Stewart et al. This content is distributed under the terms of the Creative Commons Attribution 4.0 International license.

To determine the relationship between *brlA* expression and the duration of growth inhibition, the effect of doxycycline induction on the radial growth rate of the *brlA*^I-OE^ mutant was measured (Fig. 2D). In the absence of doxycycline, the mean growth rate of the *brlA*^I-OE^ strain was slightly lower than that of wild-type A. fumigatus at 10.4 and 11.5 mm/day, respectively (90.7%). Exposure to 1.5 μg/ml of doxycycline inhibited the growth of the *brlA*^I-OE^ mutant for 2 to 3 days, after which the fungus grew but with a reduced radial growth rate (5.1 mm/day). Exposure to 20 μg/ml of doxycycline inhibited the growth of the *brlA*^I-OE^ strain for 5 to 6 days, after which the radial growth rate was 1.5 mm/day.

To determine whether nutrient availability influences the growth inhibitory effects of high-level *brlA* overexpression on precompetent hyphae, the effects of doxycycline on growth inhibition of the *brlA*^I-OE^ mutant were determined on nutrient-rich solid yeast extract-peptone-dextrose (YPD) media (Fig. S1B). In the absence of doxycycline, the growth rate of the *brlA*^I-OE^ strain was indistinguishable from wild-type A. fumigatus (24.5 and 25.0 mm/day, respectively). Doxycycline-mediated inhibition of fungal growth, however, was largely unaffected by media type. At the low dose of doxycycline, growth inhibition of the *brlA*^I-OE^ strain persisted for 2 to 3 days, followed by a reduced radial growth rate of 8.8 mm/day versus 25.1 mm/day for the wild-type strain. At the high dose of doxycycline, growth inhibition of the *brlA*^I-OE^ strain persisted for 4 to 5 days, followed by a mean radial growth rate of 3.0 mm/day versus 25.0 mm/day for wild-type A. fumigatus.

Taken together, these findings demonstrate that high levels of *brlA* overexpression inhibit the growth of precompetent A. fumigatus hyphae *in vitro*.

### Overexpression of *brlA* arrests the growth of competent *A. fumigatus* hyphae and induces conidiation in a dose-dependent manner.

**FIG 3:**
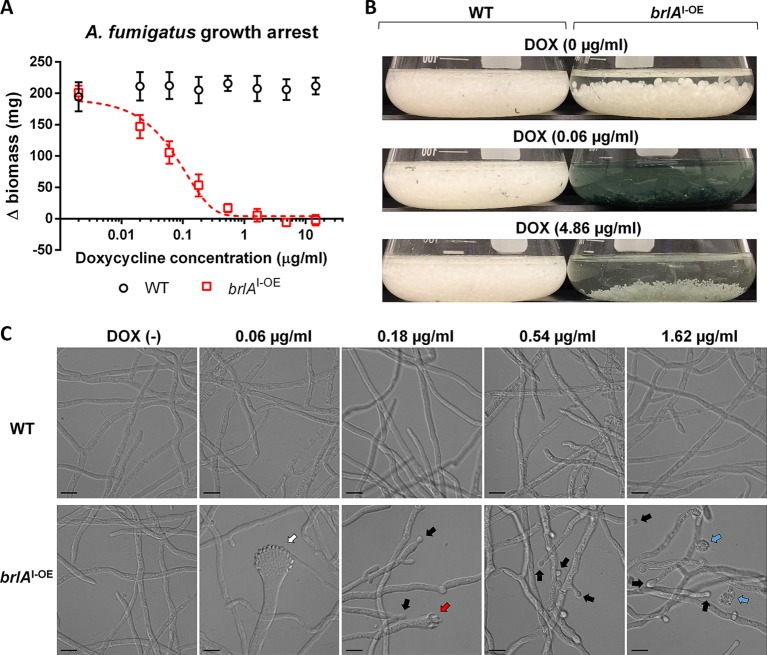
Overexpression of *brlA* in competent A. fumigatus hyphae induces dose-dependent conidiation and growth arrest. The effects of *brlA* overexpression on the growth and conidiation of competent A. fumigatus hyphae in liquid culture were assessed. (A) Change in dry weight of the parental wild-type Af293 (WT) and inducible *brlA* overexpressing (*brlA*^I-OE^) strains after treating pregrown hyphae with the indicated concentrations of doxycycline for 22 h. The combined results of three independent experiments are presented as means and standard deviations of the change in dry weight normalized to the dry weight of the same strain prior to doxycycline treatment. (B) Appearance of cultures described in panel A at the indicated concentrations of doxycycline prior to harvesting fungal biomass. (C) Representative differential interference contrast images of pregrown WT and *brlA*^I-OE^ hyphae after 4 h of exposure to the indicated concentrations of doxycycline in liquid AMM. White arrows indicate conidiophore formation, red arrows indicate atypical conidiophore formation, black arrows indicate apical and subapical budding, and blue arrows indicate hyphal rupture. Scale bars, 10 μm.

To analyze the effects of *brlA* overexpression on developmentally competent hyphae, pregrown hyphae were exposed to doxycycline then cultured for a further 22 h. The growth of wild-type A. fumigatus was unaffected by doxycycline at concentrations as high as 14.58 μg/ml (Fig. 3A). In contrast, the growth of competent hyphae of the *brlA*^I-OE^ mutant was reduced in a doxycycline dose-dependent manner, with a doxycycline MIC of 1.62 μg/ml. At this time point, hyphae of the *brlA*^I-OE^ strain, but not wild-type hyphae, began to produce macroscopically visible green conidia (Fig. 3B). This observation was most marked in *brlA*^I-OE^ cultures exposed to subinhibitory doxycycline concentrations. Microscopy of these cultures confirmed an abundance of conidiophores in doxycycline-treated *brlA*^I-OE^, while untreated *brlA*^I-OE^ cultures and wild-type cultures contained hyphae only (Fig. 3C). As with precompetent hyphae, competent *brlA*^I-OE^ cultures exposed to 0.18 μg/ml of doxycycline contained many atypical conidiophores, but these were accompanied by differentiated spherical budding structures at the hyphal tips, similar in appearance to conidia but larger in size. At 0.54 and 1.62 μg/ml of doxycycline, *brlA*^I-OE^ cultures produced fewer conidiophores and a larger number of these spherical structures. At 1.62 μg/ml, regions of the *brlA*^I-OE^ mutant hyphae appeared to have undergone lysis. Wild-type hyphae treated with doxycycline did not exhibit any of these changes. These findings suggest that low-level *brlA* overexpression reduces growth and induces conidiation in competent hyphae, whereas higher *brlA* expression leads to the formation of atypical conidiophores, altered hyphal morphology, and potentially autolysis, leading to arrested vegetative growth.

### BrlA-mediated growth inhibition requires a functional *brlA* allele.

To probe the mechanism by which competent hyphae escape growth arrest, the conidia of five *brlA*^I-OE^ breakthrough colonies (*brlA*^BT^) were isolated for analysis. PCR analysis demonstrated major deletions within the Tet-ON-*brlA* construct in 2/5 strains. In two of the remaining *brlA*^BT^ clones, reverse transcription-quantitative PCR (RT-qPCR) analysis revealed absent or attenuated doxycycline-dependent induction of *brlA*, suggesting that these strains had developed mutations within the Tet-ON-*brlA* system (Fig. S1C). The final breakthrough mutant (*brlA*^BT^ 5) exhibited minimal growth inhibition in the presence of doxycycline despite the induction of high levels of *brlA* expression (Fig. S1C and S2A). When grown on solid media, colonies of the *brlA*^BT^ 5 strain were more compact and exhibited reduced conidiation in the presence of doxycycline compared to doxycycline-free conditions and wild-type controls (Fig. S2B). Sequencing of the Tet-ON-*brlA* ORF of the *brlA*^BT^ 5 strain identified a nucleotide insertion at position 1083, leading to a frameshift mutation and the production of an altered BrlA protein (BrlA^BT^), predicted to contain 24 altered amino acid residues at the C terminus and truncated by 42 amino acid residues (Fig. S2C).

10.1128/mBio.03202-19.2 FIG S2 An inducible *brlA* overexpression breakthrough mutant maintains growth but has impaired conidiation in response to doxycycline *in vitro*. (A) Radial growth of the parental wild-type Af293 (WT) strain, the inducible *brlA* overexpressing (*brlA*^I-OE^) mutant, and a spontaneous *brlA*^I-OE^ breakthrough isolate (*brlA*^BT^ 5) on solid AMM media after exposure to 20 μg/ml doxycycline. Each data point represents the mean and the standard deviation of 3 biological replicates each with two to three replicates. (B) Appearance of the WT, *brlA*^I-OE^ and *brlA*^BT^ 5 strains after 3 days of growth under the conditions described in panel A. (C) Amino acid sequences of the WT strain BrlA protein (BrlA) and the *brlA*^BT^ 5 mutant BrlA protein (BrlA^BT^) as determined by Sanger sequencing of genomic DNA and *in silico* translation to protein. Blue highlighting indicates region unique to wild-type BrlA and yellow highlight indicates the mutated region unique to BrlA^BT^. Download FIG S2, TIF file, 1.5 MB. Copyright © 2020 Stewart et al. 2020 Stewart et al. This content is distributed under the terms of the Creative Commons Attribution 4.0 International license.

Bioinformatics analyses suggest that BrlA is a canonical C2H2 transcription factor that contains two zinc finger (ZnF) motifs, residues 316 to 340 (ZnF-I) and 346 to 371 (ZnF-II). Each zinc finger has the consensus sequence X_2_-Cys-X_2,4_-Cys-X_12_-His-X_3,4,5_-His (21–25) (Fig. S3A). Sequence alignment of BrlA and BrlA^BT^ reveals that the first 361 residues are conserved between the two proteins, including the ZnF-I motif (26) (Fig. S3B). To understand the structural consequences of the frameshift mutation, homology models of BrlA_300–426_ and BrlA^BT^_300–385_ were created using Phyre^2^ (27) (Fig. S3C). The structural model of BrlA_300–426_ was based on the structure of Krueppel-like factor 10. The two ZnF motifs in BrlA form classical ββα folds. Zinc coordination by ZnF motifs requires two conserved cysteines and histidines, located on one end of the β-sheet and the C terminus of the DNA recognition helix, respectively (28). Residues at positions –1, +2, +3, and +6 of the α-helix are hypothesized to make contacts with nucleotides (28). The amino acid sequence of BrlA^BT^ is altered after D361, which is located at the N terminus of ZnF-II recognition helix. H366P and H371P mutations at the C terminus of the recognition helix would abrogate zinc coordination by ZnF-II. In addition, the C365A, and charge switch mutation, N362K, at the +6 and +3 positions, respectively, are predicted to affect binding of the recognition helix to DNA. Residues 372 to 426 of BrlA are predicted to be partially disordered and could not be modeled accurately by Phyre^2^ (27). The role of this region in DNA binding and/or transcriptional activation (29) is unclear. This model suggests that BrlA^BT^_300–385_ is likely impaired in its ability to bind DNA and mediate transcriptional regulation.

10.1128/mBio.03202-19.3 FIG S3 A frameshift mutation in *brlA* ORF disrupts the second zinc finger motif. (A) Linear schematics of BrlA and the BrlA^BT^ mutant. Two zinc finger motifs (yellow diamonds labelled ZnF) are located between residues 316 to 340 and 346 to 371 of BrlA. Residues 362 to 385, highlighted in red, are mutated in BrlA^BT^ as a result of the frameshift mutation. (B) Sequence alignment of residues 346 to 371 of BrlA and BrlA^BT^ with predicted secondary structure below (27, 28, 52, 53, 64). Cysteine and histidine residues predicted to coordinate a zinc atom are colored orange. Residues that differ between BrlA and BrlA^BT^ are highlighted in red. Residues predicted to contact DNA are numbered. (C) Structural model of BrlA_300–426_, homologous to the human Krueppel-like factor 10 (PDB ID 2EPA; confidence, 99.7%; coverage, 56%; identity, 37%) predicted using Phyre^2^ (27). Left: BrlA^316-371^ shown in cartoon representation with transparent surface. ZnF-I is colored cyan and ZnF-II is colored magenta. Cysteines and histidines predicted to coordinated zinc are shown as sticks. Images were rendered using PyMOL (v2.3.2) Right: Cartoon representation of ZnF-II of BrlA and BrlA^BT^ with side chains predicted to bind zinc and contact DNA shown as sticks. Download FIG S3, TIF file, 1.0 MB. Copyright © 2020 Stewart et al. 2020 Stewart et al. This content is distributed under the terms of the Creative Commons Attribution 4.0 International license.

Overall, the finding that all breakthrough strains had either lost inducible *brlA* expression or acquired mutations within the *brlA* ORF confirms that doxycycline-mediated growth inhibition of the *brlA*^I-OE^ mutant is a direct consequence of *brlA* overexpression and not an off-target effect of the Tet-ON system.

### Overexpression of *brlA* attenuates the virulence of *A. fumigatus* in an invertebrate and a mouse model of invasive aspergillosis.

To determine whether *brlA* overexpression could reduce virulence *in vivo*, larvae of the Galleria mellonella moth were infected with conidia of wild-type or *brlA*^I-OE^ mutant A. fumigatus with or without 400 μg/ml doxycycline. Five days after infection, 47% of the *brlA*^I-OE^-infected, doxycycline-treated larvae had succumbed to infection compared to 84% of the untreated *brlA*^I-OE^-infected larvae (Fig. S4). Doxycycline-treated and untreated larvae infected with wild-type A. fumigatus displayed similarly high mortality rates (100 and 94%, respectively). These findings suggest that upregulation of *brlA* significantly reduces A. fumigatus virulence in an invertebrate model of IA.

10.1128/mBio.03202-19.4 FIG S4 Doxycycline attenuates the virulence of the inducible *brlA* overexpression strain of A. fumigatus in an invertebrate model of invasive aspergillosis. Survival of G. mellonella larvae infected with conidia of the parental wild-type Af293 (WT) and the inducible *brlA* overexpressing (*brlA*^I-OE^) strains in the presence of 3 μg doxycycline. *n* = at least 32 larvae per group from three independent experiments. *, *P < *0.05 [Mantel-Cox log rank test comparing *brlA*^I-OE^ DOX (–) to WT DOX (–)]; ****, *P < *0.0001 [Mantel-Cox log rank test comparing *brlA*^I-OE^ DOX (+) to *brlA*^I-OE^ DOX (–)]. Download FIG S4, TIF file, 0.2 MB. Copyright © 2020 Stewart et al. 2020 Stewart et al. This content is distributed under the terms of the Creative Commons Attribution 4.0 International license.

The effects of *brlA* overexpression in a neutropenic mouse model of invasive aspergillosis were also assessed (30, 31). To confirm that doxycycline treatment resulted in *brlA* overexpression in the *brlA*^I-OE^ mutant during infection, mice infected with the *brlA*^I-OE^ strain were treated with doxycycline, and *brlA* expression was quantified using RT-qPCR (Fig. S5A). Treatment of *brlA*^I-OE^-infected mice with a single dose of doxycycline resulted in a 4.2-fold higher level of *brlA* expression within their lungs relative to doxycycline-free controls. No expression of *brlA* was detectable in mice infected with wild-type A. fumigatus.

10.1128/mBio.03202-19.5 FIG S5 Doxycycline treatment induces *brlA* overexpression in the *brlA*^I-OE^ strain during A. fumigatus pulmonary infection. (A) Expression of *brlA* in the parental wild-type Af293 (WT) and inducible *brlA*-overexpressing (*brlA*^I-OE^) strains of A. fumigatus as measured by RT-qPCR 3 h after treatment of infected mice with a single intraperitoneal injection of 25 mg/kg of doxycycline. Gene expression was normalized to the endogenous reference gene *tef1* and expressed as the fold change relative to the inducible *brlA*-overexpressing strain in the absence of doxycycline. The data are presented as the mean and the standard error of 4 biological replicates, each performed in triplicate. *, *P < *0.05 [two-tailed Student t-test comparing *brlA*^I-OE^ DOX (+) to *brlA*^I-OE^ DOX (–) at the same time point]. ND, transcript not detected. (B and C) Serum doxycycline concentration in neutropenic BALB/c mice infected with the indicated A. fumigatus strains and treated with either daily intraperitoneal injections of 10 mg/kg doxycycline (B) or 100 mg/kg doxycycline every 12 h via oral gavage (C) and as determined using uHPLC-MS. Data represent the median of three mice from a single experiment. Download FIG S5, TIF file, 0.1 MB. Copyright © 2020 Stewart et al. 2020 Stewart et al. This content is distributed under the terms of the Creative Commons Attribution 4.0 International license.

**FIG 4:**
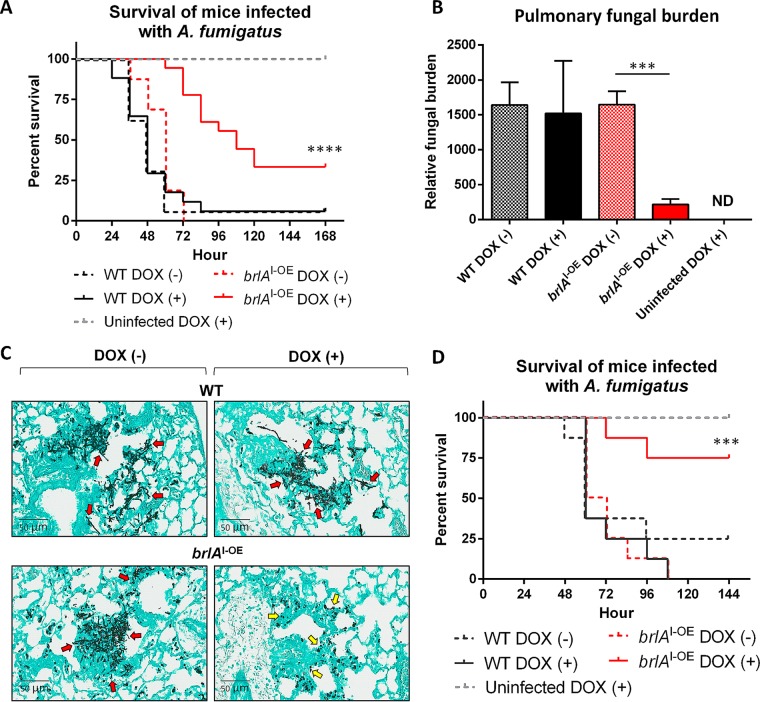
Overexpression of *brlA* attenuates the virulence of A. fumigatus in a mouse model of invasive aspergillosis. (A) Survival of neutropenic BALB/c mice receiving daily intraperitoneal injections of 10 mg/kg doxycycline and infected with A. fumigatus conidia of the parental wild-type Af293 (WT) or the inducible *brlA* overexpressing (*brlA*^I-OE^) strains. *n* = at least 16 mice per group from two independent experiments. ****, *P < *0.0001 [Mantel-Cox log rank test comparing *brlA*^I-OE^ DOX (+) to *brlA*^I-OE^ DOX (–)]. (B) Pulmonary fungal burden of mice infected and treated as in panel A at 36 h postinfection, as determined by pulmonary galactomannan quantification. *n* = 8 mice per group. ***, *P < *0.001 [Wilcoxon rank sum test comparing *brlA*^I-OE^ DOX (+) to *brlA*^I-OE^ DOX (–)]. (C) Gomori methenamine-silver-stained lung sections from mice infected and treated as in panel A at 36 h postinfection. Red arrows indicate hyphal filaments, and yellow arrows indicate swollen conidia and small hyphae. (D) Survival of neutropenic BALB/c mice receiving 100 mg/kg doxycycline every 12 h via oral gavage and infected with conidia of the WT or *brlA*^I-OE^ strains. *n* = 8 mice per group. ***, *P < *0.001 [Mantel-Cox log rank test comparing *brlA*^I-OE^ DOX (+) to *brlA*^I-OE^ DOX (–)].

Since doxycycline-induced *brlA* overexpression was most effective at inhibiting the growth of precompetent A. fumigatus hyphae *in vitro*, mice were treated with doxycycline prior to infection and throughout the course of the experiment. Mice infected with the *brlA*^I-OE^ strain and treated with doxycycline exhibited improved survival compared to untreated mice (33% versus 0%, respectively; Fig. 4A), as well as treated and untreated mice infected with wild-type A. fumigatus (6% in both groups). Pulmonary fungal burden levels were significantly lower in doxycycline-treated *brlA*^I-OE^-infected mice compared to untreated animals or mice infected with wild-type A. fumigatus with or without doxycycline treatment (Fig. 4B). Pulmonary histopathology examination was consistent with the survival and pulmonary fungal burden determination studies. Gomori methenamine-silver staining of lung sections revealed that the lungs of doxycycline-treated *brlA*^I-OE^-infected mice contained fewer fungal lesions than the other experimental groups, and these lesions were largely composed of swollen conidia and short hyphae (Fig. 4C). In contrast, untreated *brlA*^I-OE^-infected mice or mice that were infected with wild-type A. fumigatus with or without doxycycline treatment had more and larger pulmonary lesions containing long hyphae. No significant immune cell infiltration surrounding fungal lesions was observed in any of the groups of infected mice, likely reflecting the profound neutropenia induced in these animals. Collectively these results suggest that overexpression of *brlA* early in fungal infection attenuates fungal growth and virulence in a mouse model of invasive aspergillosis.

Although treatment with doxycycline increased the survival of *brlA*^I-OE^-infected mice, the majority of mice eventually succumbed to infection. We therefore hypothesized that the concentrations of doxycycline at the site of infection may be near or below the *brlA*^I-OE^ strain MIC, permitting fungal growth. To test this hypothesis, the serum concentrations of doxycycline in infected mice were quantified using ultra-high-performance liquid chromatography coupled to mass spectrometry (uHPLC-MS/MS) at 36 h postinfection. Median serum concentrations of doxycycline in *brlA*^I-OE^-infected and wild-type-infected mice were 290 and 392 ng/ml, respectively (Fig. S5B), confirming that the doxycycline concentrations were below the *brlA*^I-OE^ strain MIC of 540 ng/ml during much of the infection course, likely allowing continued fungal growth.

To determine whether increased serum doxycycline levels could improve survival of mice infected with the *brlA*^I-OE^ mutant, the survival experiment was repeated with a 10-fold higher dose of doxycycline administered every 12 h via oral gavage. This regimen resulted in significantly higher median serum doxycycline concentrations in *brlA*^I-OE^- and wild-type-infected mice (5,564 and 4,194 ng/ml, respectively; Fig. S5C). Higher-dose doxycycline treatment resulted in an improved survival rate of mice infected with the *brlA*^I-OE^ mutant (75% versus 0% in treated versus untreated mice, Fig. 4D) compared to lower-dose therapy. These results illustrate a dose-dependent relationship between doxycycline exposure and mortality *in vivo*, supporting the hypothesis that *brlA* overexpression reduces A. fumigatus virulence.

### RNA sequencing of *brlA*-overexpressing precompetent *A. fumigatus* hyphae reveals altered gene expression patterns associated with signaling, amino acid, and carbohydrate metabolism.

To explore the mechanisms by which high-level *brlA* overexpression mediates growth inhibition of precompetent A. fumigatus hyphae and attenuates virulence, an RNA sequencing (RNA-seq)-based approach was used. Pre-competent hyphae of the *brlA*^I-OE^ mutant and wild-type parent were exposed to doxycycline for 30 min before RNA extraction.

**FIG 5:**
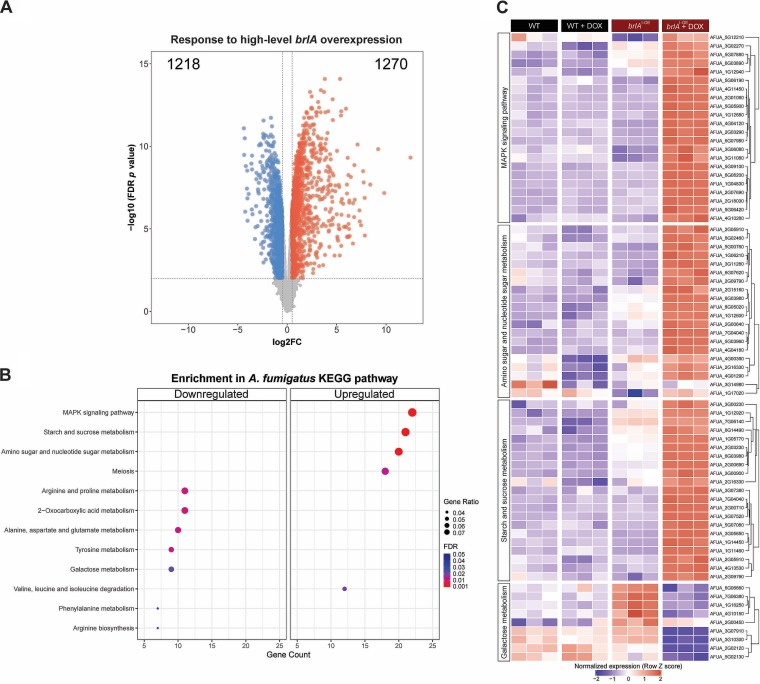
High-level *brlA* overexpression in precompetent A. fumigatus hyphae results in significant changes in the patterns of gene transcription. Differentially expressed genes (DEGs) were examined for the contrast between precompetent hyphae of the inducible *brlA* overexpressing (*brlA*^I-OE^) and parental wild-type Af293 (WT) strains after 30 min exposure to 20 μg/ml doxycycline as measured by RNA sequencing. (A) Volcano plot highlighting downregulated (blue) and upregulated (red) genes with a log_2_(fold change) (log_2_FC) > 0.5 and an FDR *P < *0.01. (B) Enrichment analysis of DEGs from panel A using the Kyoto Encyclopedia of Genes and Genomes (KEGG) cataloged pathways for A. fumigatus with FDR *P < *0.05. (C) Hierarchical clustered heatmap for the DEGs in three of the upregulated and one of the downregulated KEGG-enriched pathways from panel B.

Consistent with our *in vitro* RT-qPCR studies, the *brlA*^I-OE^ mutant exhibited baseline overexpression of *brlA*, which increased dramatically after doxycycline treatment. In comparison, the wild-type strain displayed only low levels of *brlA* transcripts in either condition (Fig. S6A). A principal-component analysis (PCA) demonstrated clustering of the biological replicates within groups and differentiated the doxycycline-treated *brlA*^I-OE^ mutant from the other groups (Fig. S6B). To identify gene expression changes that could be attributed specifically to high-level *brlA* overexpression, the RNA-seq analysis was performed in two steps. First, the effect of doxycycline on A. fumigatus gene expression was quantified independently for the wild-type and *brlA*^I-OE^ strains. Next, the effects of doxycycline treatment on the *brlA*^I-OE^ mutant transcriptome were compared to those observed in the wild-type strain, to identify transcriptional changes that could be attributed specifically to high-level *brlA* overexpression. The gene expression profile of the *brlA*^I-OE^ mutant was strongly affected by high-level *brlA* overexpression, with 1,270 genes being upregulated and 1,218 genes downregulated using a cutoff log_2_(fold change) of >0.5 relative to the *brlA*^I-OE^ mutant at baseline (Fig. 5A). The expression levels of the majority of doxycycline response genes identified in the *brlA*^I-OE^ mutant were not significantly differentially expressed between the untreated *brlA*^I-OE^ mutant and wild-type parent (Fig. S6C), indicating that high-level rather than low-level *brlA* overexpression is required to mediate widespread transcriptomic changes in precompetent A. fumigatus hyphae.

10.1128/mBio.03202-19.6 FIG S6 Doxycycline-induced high-level *brlA* overexpression in precompetent A. fumigatus hyphae leads to widespread transcriptomic changes that are not observed in hyphae with low-level *brlA* overexpression. (A) Number of *brlA* copies per million reads sequenced (cpm) and (B) Principal component analysis (PCA) of RNA transcript abundance in precompetent hyphae of the parental wild-type Af293 (WT) and inducible *brlA*-overexpressing (*brlA*^I-OE^) strains in the presence or absence of doxycycline. (C) Log_2_(fold change) (log_2_FC) density of the downregulated and upregulated genes identified in the doxycycline-treated *brlA*^I-OE^ mutant relative to the untreated controls (red) and the log_2_FC density of these same genes in the untreated *brlA*^I-OE^ mutant relative to WT (gray). Hyphae of the indicated strains of A. fumigatus were exposed to 20 μg/ml doxycycline for 30 min before RNA processing and transcriptome analysis as measured by RNA sequencing. For all comparisons genes with a log_2_FC > 0.5 and with an FDR of *P < *0.01 were considered differentially expressed. Download FIG S6, TIF file, 0.1 MB. Copyright © 2020 Stewart et al. 2020 Stewart et al. This content is distributed under the terms of the Creative Commons Attribution 4.0 International license.

To identify the pathways most affected by high-level *brlA* overexpression, a gene set enrichment analysis was performed. A list of differentially regulated genes with a false discovery rate (FDR) of *P* < 0.01 in the doxycycline-treated *brlA*^I-OE^ mutant was compared to Kyoto Encyclopedia of Genes and Genomes (KEGG) catalogued pathways (Fig. 5B). The representative categories of upregulated genes included mitogen-activated protein kinase (MAPK) signaling, meiosis, and processes of carbohydrate and nitrate metabolism (Fig. 5B and C). Consistent with the role of *brlA* in A. fumigatus conidiation, several upregulated genes within the MAPK signaling and meiosis categories encode key regulators of conidiation, including the transcriptional activator of conidiation AbaA (AFUA_1G04830) and the APSES family protein StuA (AFUA_2G07900) (Fig. 5C; Tables S1 and S2). Conversely, very few conidiation-related genes were upregulated in the *brlA*^I-OE^ mutant at baseline (Fig. 5C; Tables S1, S2, and S3), consistent with the observation that doxycycline was required to induce conidiation in the *brlA*^I-OE^ mutant. The representative categories of downregulated genes were enriched exclusively in processes of metabolism, including several amino acids and the carbohydrate galactose (Fig. 5B and C). Overexpression of *brlA* also resulted in altered regulation of the genes governing the biosynthesis of galactosaminogalactan (GAG) and trehalose, two other carbohydrates known to play a role in A. fumigatus virulence (32–35). Genes involved in the biosynthesis of GAG were downregulated in response to high-level *brlA* overexpression, whereas those involved in trehalose biosynthesis were upregulated (Fig. 5C; Tables S1, S2, and S3). Taken as a whole, these findings suggest that high-level *brlA* overexpression not only leads to significant shifts in cell signaling and amino acid metabolism but may also result in changes in the production of carbohydrates that modulate virulence.

10.1128/mBio.03202-19.7 TABLE S1 Complete list of genes identified by RNA sequencing of precompetent wild-type and inducible *brlA* overexpression strain hyphae in the presence or absence of doxycycline. Download Table S1, XLSX file, 3.2 MB. Copyright © 2020 Stewart et al. 2020 Stewart et al. This content is distributed under the terms of the Creative Commons Attribution 4.0 International license.

10.1128/mBio.03202-19.8 TABLE S2 Enriched KEGG pathways in precompetent A. fumigatus hyphae with high-level *brlA* overexpression. Download Table S2, XLSX file, 0.04 MB. Copyright © 2020 Stewart et al. 2020 Stewart et al. This content is distributed under the terms of the Creative Commons Attribution 4.0 International license.

### Overexpression of *brlA* in precompetent hyphae leads to alterations in the production of two carbohydrates that play a role in *A. fumigatus* virulence.

**FIG 6:**
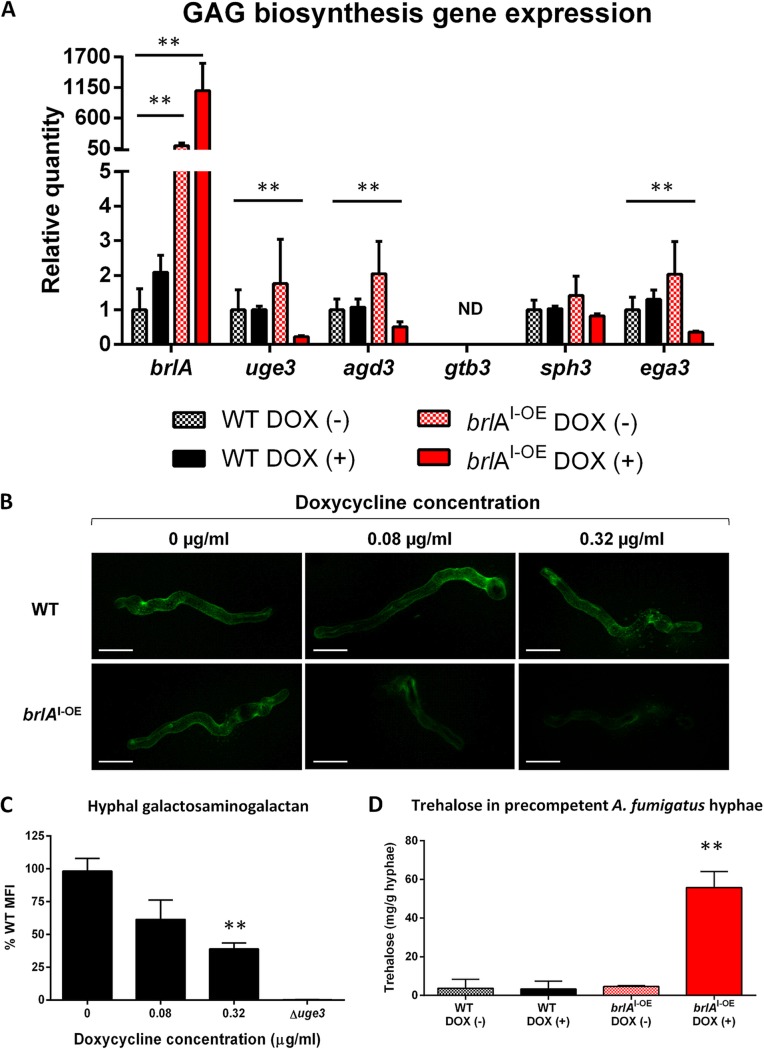
*brlA* overexpression in precompetent A. fumigatus hyphae leads to the altered production of two virulence factor carbohydrates. (A) Gene expression levels of genes (*uge3*, *agd3*, *gtb3*, *sph3*, and *ega3*) that encode for proteins involved in the biosynthesis of GAG, as measured by RT-qPCR in hyphae of the parental wild-type Af293 (WT) and the inducible *brlA* overexpressing (*brlA*^I-OE^) strains after exposure to 20 μg/ml doxycycline for 30 min. Gene expression was normalized to the endogenous reference gene *tef1* and is presented as the fold change relative to wild-type expression in the absence of doxycycline. The data are presented as the means and standard errors of four biological replicates, each performed in triplicate. **, *P < *0.0001 [two-tailed Student *t* test. *brlA*^I-OE^ DOX (–) and *brlA*^I-OE^ DOX (+) were compared to wild-type DOX (–) at the same time point]. ND, transcript not detected. (B) Representative confocal images of WT and *brlA*^I-OE^ hyphae stained with a GAG-specific fluorescein-tagged soybean agglutinin lectin (SBA-FITC) after 8 h of exposure to the indicated concentrations of doxycycline. Scale bars, 10 μm. (C) Quantification of the MFIs of *brlA*^I-OE^ hyphae normalized to the MFI of the WT strain grown under the same conditions as in panel B and compared to hyphae of the GAG-deficient strain of A. fumigatus (Δ*uge3*) as a negative control. The data are presented as the percent WT MFI and the standard errors from three independent experiments, each with a minimum of eight hyphae per condition. (D) Quantification of trehalose in precompetent hyphae of the WT and *brlA*^I-OE^ strains of A. fumigatus grown in the presence or absence of 0.08 μg/ml doxycycline. The data are presented as the means and standard deviations of three biological replicates. **, *P < *0.01 [two-tailed Student *t* test for *brlA*^I-OE^ DOX (+) compared to wild-type DOX (–) at the same time point].

The results of the RNA-seq experiments suggest that *brlA* overexpression decreases GAG biosynthesis (34–40). To validate our RNA-seq results, RT-qPCR was used to test the effects of doxycycline treatment on the expression of GAG biosynthetic genes in hyphae of the *brlA*^I-OE^ mutant. Consistent with the RNA-seq results, significant doxycycline-dependent downregulation of GAG cluster genes *uge3*, *agd3*, and *ega3* were observed in the *brlA*^I-OE^ strain (Fig. 6A). The expression of *gtb3* was below the limit of detection under all conditions tested. No effects of doxycycline treatment on the expression of GAG cluster genes were observed in the wild-type parent. In the presence of subinhibitory concentrations of doxycycline, hyphae of the *brlA*^I-OE^ mutant exhibited reduced levels of GAG, as determined by GAG-specific fluorescein-tagged soybean agglutinin lectin (SBA-FITC) staining (Fig. 6B). The mean fluorescence intensity (MFI) of the *brlA*^I-OE^ strain was significantly reduced in the presence of doxycycline relative to the wild-type parent under the same conditions (38.9%), and a trend toward a reduced MFI was observed in the *brlA*^I-OE^ strain even at low concentrations of doxycycline (61.4% relative to the wild type; Fig. 6B and C). GAG staining of wild-type hyphae was unaffected by doxycycline. Taken together, these results demonstrate that *brlA* overexpression leads to the reduced synthesis of GAG, which may contribute to attenuated virulence.

Our RNA-seq studies also suggested that *brlA* overexpression upregulates hyphal trehalose synthesis. Consistent with this observation, doxycycline treatment of *brlA*^I-OE^ mutant hyphae resulted in a >12-fold increase in trehalose content compared to the wild-type parent or the untreated *brlA*^I-OE^ strain (55.8 versus 3.7 versus 4.6 mg trehalose/g respectively; Fig. 6D). Doxycycline treatment had no effect on the wild-type hyphal trehalose. As an inverse relationship between trehalose synthesis and virulence has been reported in A. fumigatus (32, 33), this increase in hyphal trehalose synthesis may also contribute to *brlA*-mediated attenuation of virulence.

## DISCUSSION

In the present study, we demonstrate that the expression of a single transcriptional regulator, *brlA*, is sufficient to activate the conidiation pathway in A. fumigatus, inhibit vegetative growth *in vitro* and reduce virulence in two *in vivo* models of invasive *Aspergillus* infection. RNA-seq and phenotypic studies suggest that multiple *brlA*-dependent mechanisms likely contribute to this reduced fungal growth and attenuation of virulence.

The results of our RNA-seq studies provide evidence for *brlA*-dependent dysregulation of the mitotic cell cycle, including overexpression of the genes encoding a protein tyrosine phosphatase NimT/Mih1, as well as the 14-3-3 family protein ArtA. NimT is a Cdc25-type phosphatase that is an essential regulator of cell cycle progression and growth in A. nidulans, and a *nimT-*deficient strain undergoes late G_2_- and M-phase arrest shortly after germination (41, 42). ArtA plays a role in regulating germ tube formation and hyphal morphogenesis in A. nidulans. Overexpression of *artA* in A. nidulans results in abnormal germ tube formation and hyphal branching, as well as significantly reduced colony size (41). Strong upregulation of these two genes in A. fumigatus in response to *brlA* overexpression likely indicates dysfunctional cell cycle control, leading to defects in mitosis and growth.

The overexpression of A. fumigatus *brlA* also led to a fundamental switch in transcription of metabolic genes, with alterations in primary carbon metabolism pathways, including glycolysis, gluconeogenesis, the tricarboxylic acid cycle, and the glyoxylate cycle (Tables S1 and S3). Not surprisingly, many of these metabolic pathways are also differentially regulated in A. nidulans following the induction of asexual development (43), demonstrating the highly conserved and largely BrlA-mediated regulation of metabolism during conidiation in these two *Aspergillus* spp.

The results of our RNA-seq experiments also revealed *brlA*-dependent changes in expression of high-osmolarity glycerol response (HOG)-MAPK and protein kinase A (PKA) pathway genes, including *sakA*, *mpkC*, and *pkaR*. The HOG-MAPKs SakA and MpkC regulate PKA signaling and SakA physically interacts with PkaC and PkaR in a complex to increase the phosphorylation state and signaling activity of PKA in A. fumigatus during osmotic or cell wall stressing conditions (44, 45). Conidiation is an effective propagation strategy that allows the fungus to escape from unfavorable conditions, and mechanisms that can sense and respond to cell wall stress, such as the HOG-MAPK and PKA pathways, are therefore likely to interact with those involved in the processes of conidiation. The results of our RNA sequencing suggest this interaction may occur in large part through the activity of BrlA.

Altered HOG-MAPK and PKA signaling in response to *brlA* overexpression may also explain some of the large-scale transcriptional changes observed in sugar metabolism. In A. fumigatus HOG-MAPK and PKA signaling are essential for carbohydrate mobilization and metabolism, including glucose, glycogen and trehalose, maintenance of cell wall morphology, and protection against cell wall-perturbing agents (44). We observed increased expression of a trehalose-6-phosphate phosphatase encoding gene *orlA* and a striking upregulation in hyphal trehalose in response to *brlA* overexpression. During increased trehalose biosynthesis, glucose-6-phosphate would likely become limiting, and this is evidenced at the transcriptomic level by altered expression of several hexokinase-encoding genes. Increased trehalose biosynthesis also likely reflects a diversion of carbon away from other processes such as cell wall and extracellular matrix production. Our findings support this hypothesis, since *brlA-*overexpressing hyphae exhibited increased expression of genes encoding chitin- and β-glucan-degrading enzymes (46), reduced expression of GAG biosynthesis genes, and lower levels of hyphal GAG. Since both increased trehalose production and reduced GAG production are associated with impaired virulence, it is likely that these changes in carbohydrate metabolism contribute to improved survival of mice infected with *brlA*-overexpressing A. fumigatus.

Taken as a whole, these results suggest that *brlA* overexpression results in multiple changes in carbohydrate and cell wall composition that lead to growth arrest. These observations are consistent with the histopathological findings of poor hyphal growth of *brlA*-overexpressing A. fumigatus *in vivo*. Interestingly, the survival of neutropenic mice infected with *brlA*-overexpressing A. fumigatus was similar to that reported during infection with the α-glucan-deficient triple *ags* mutant (65). This strain also exhibits a profound alteration in cell wall composition and fails to germinate *in vivo*. These similarities highlight the critical importance of cell wall architecture during the pathogenesis of A. fumigatus invasive infection.

One concern regarding the use of *brlA* overexpression as a therapeutic strategy is the potential adverse effects of inducing conidiation during infection. However, conidiophores were not observed within the lungs of any mice infected with the inducible *brlA* overexpression strain. The absence of conidiation in mice, despite serum doxycycline levels falling below the MIC of 540 ng/ml, may indicate an accumulation of doxycycline within lung tissues, as has been reported in rats (47). Alternatively, since the conidiation of A. fumigatus is rarely observed during invasive infection (10), other host or fungal factors may actively suppress conidiation *in vivo*.

Although *brlA* induction was highly effective in inhibiting precompetent hyphal growth, escape from *brlA*-mediated growth arrest was more rapid in competent hyphae. Breakthrough strains exhibited spontaneous mutations within the Tet-ON-*brlA* system or mutations within the *brlA* ORF which would be predicted to reduce BrlA activity. These findings suggest that competent hyphae are more prone to developing resistance mutations permitting vegetative growth. These observations suggest that while small molecule activators of conidiation may provide useful agents for the prevention of invasive aspergillosis in high-risk individuals, their use for the treatment of established infection may be limited by the emergence of resistance.

The results of this study provide evidence that induction of the conidiation pathway, through the activation of *brlA*, is novel approach to reduce A. fumigatus vegetative growth *in vitro* and virulence *in vivo*. These studies identify the conidiation pathway as a novel target for antifungal therapeutics and provide a foundation for further studies to identify *brlA*-inducing small molecules that may one day be used for prevention of invasive A. fumigatus infection.

## MATERIALS AND METHODS

### Fungal strains and growth conditions.

A. fumigatus strain Af293 (wild-type) was used to construct the inducible *brlA* overexpression strain (*brlA*^I-OE^). All strains were routinely cultured at 37°C on yeast extract-peptone-dextrose (YPD) media (BD Difco). All *in vitro* assays were performed at 37°C in either YPD or *Aspergillus* minimal media (AMM) (48) supplemented with 3× trace elements and 1.5% agar for solid medium conditions.

### Modification of tetracycline-inducible gene expression vectors.

The optimized Tet-ON plasmid pJW128 (15; a gift from Robert Cramer, Geisel School of Medicine) contains the Tet-ON system, consisting of a doxycycline-responsive reverse transactivator gene (*rtTA*) fused to a strong viral activation domain, a transactivator-response element (*TetO_7_*) embedded within a minimal *oliC* promoter (*Pmin*) upstream of the gene of interest (49), and a resistance cassette for pyrithiamine. The *brlA* ORF was amplified from wild-type A. fumigatus genomic DNA (gDNA) and fused to the A. nidulans tryptophan biosynthesis gene (*trpC*) terminator via PCR, generating *brlA-TtrpC*. The *brlA-TtrpC* PCR product was subcloned into the blunt cloning vector pCR-Blunt-II-TOPO (Invitrogen), generating pCR-*brlA-TtrpC*. Finally, this *brlA-TtrpC* fragment was subcloned by PmeI and BsuCCCLXI digestion into pJW128, generating the final vector pJW128-*brlA*-*TtrpC* (Tet-ON-*brlA*). Final plasmids were validated using Sanger sequencing.

### Transformation of *A. fumigatus* wild-type strain (Af293).

Transformation of the A. fumigatus wild-type strain (Af293) was performed as previously described (50). Antifungal-resistant transformants were selected using 0.5 μg/ml pyrithiamine (Sigma). Verification of the presence of the linear Tet-ON-*brlA* construct and the absence of a circular autonomously replicating Tet-ON-*brlA* plasmid within pyrithiamine-resistant transformants was accomplished by PCR analysis of genomic DNA.

### Quantitative real-time PCR analysis.

Quantification of mRNA expression was performed using SsoAdvanced Universal SYBR green supermix (Bio-Rad) and a 7300 real-time PCR system (Applied Biosystems). For *in vitro* experiments, strains were grown at 37°C in AMM broth for 18 h, followed by exposure to 1.5 or 20 μg/ml doxycycline. Fungal RNA was isolated using the Nucleospin RNA plant kit (Macherey-Nagel) and reverse transcribed into cDNA using the QuantiTect reverse transcription kit (Qiagen), and then RT-PCR was performed as previously described (51) using the primers listed in Table S4.

10.1128/mBio.03202-19.9 TABLE S3 Selected differentially expressed genes and their associated pathways in precompetent A. fumigatus hyphae with high-level *brlA* overexpression. Download Table S3, XLSX file, 0.02 MB. Copyright © 2020 Stewart et al. 2020 Stewart et al. This content is distributed under the terms of the Creative Commons Attribution 4.0 International license.

10.1128/mBio.03202-19.10 TABLE S4 Primers used for the construction and screening of a doxycycline-inducible *brlA* overexpression A. fumigatus mutant. Download Table S4, XLSX file, 0.01 MB. Copyright © 2020 Stewart et al. 2020 Stewart et al. This content is distributed under the terms of the Creative Commons Attribution 4.0 International license.

### Growth kinetic assays *in vitro*.

For growth inhibition analyses of precompetent A. fumigatus, 1 × 10^4^ conidia were cultured for 20 h in AMM containing doxycycline at the indicated concentrations. Growth inhibition was assessed by staining the resulting biomass with crystal violet and quantifying the optical density at 600 nm as previously described (37). Changes to precompetent fungal morphology were assessed by inoculating 1 × 10^4^ conidia per well in 24-well plates containing sterile coverslips and AMM supplemented with the indicated concentrations of doxycycline. At 20 h of growth, the coverslips were washed and fixed in 4% paraformaldehyde and then mounted and imaged using a LSM780 laser scanning confocal microscope (Zeiss) with a 63× oil objective lens. Images were processed using ZEN blue edition software (Zeiss). For precompetent shaking cultures, 1 × 10^6^ conidia/ml were grown in AMM containing the indicated concentrations of doxycycline for 48 h. Solid medium growth assays were performed on AMM and YPD media supplemented with indicated concentrations of doxycycline. A total of 100 conidia were point inoculated onto solid media, and the diameters of fungal colonies were measured for 7 days.

For assays of competent hyphae, 1 × 10^6^ conidia/ml were incubated in AMM for 18 h and then treated with doxycycline. Images were acquired, and the fungal biomass was lyophilized for the determination of dry biomass. Solid medium growth assays were performed as with precompetent hyphae but with the addition of doxycycline after 24 h of growth. The morphology of competent hyphae was assessed as with precompetent hyphae with the addition of doxycycline at 18 h of growth.

### Protein modeling.

The amino acid sequences of A. fumigatus BrlA and the BrlA^BT^ mutant were submitted to BLASTP and HMMER servers to identify known domains and sequence features (21, 23). Zinc finger motif predictions were performed using the C2H2 position weight matrix (PMW) server and PROSITE database (22, 24, 25). ClustalOmega was used for multiple sequence alignments (26), and the PSIPRED, JPred4, and Phyre^2^ servers were used for structural prediction (27, 52, 53). PrDOS was used to predict disordered regions (29).

### *Galleria mellonella* larva infection model.

Galleria mellonella larvae (Magazoo, Montreal, Quebec, Canada) were infected with A. fumigatus conidia as described previously (54). Briefly, conidia were resuspended at a concentration of 2 × 10^8^ conidia/ml in phosphate-buffered saline (PBS) with or without 400 μg/ml doxycycline. Larvae were infected with 1.5 × 10^6^ conidia with or without 3 μg of doxycycline by injection of the last proleg using a Hamilton 25-μl glass gas-tight syringe (1702RN) with a 33G gas chromatography needle (33/1.5′/3). Uninfected controls received PBS with doxycycline. Larvae were incubated in the dark at 37°C for 5 days. Death was confirmed by a combination of melanization and a lack of movement.

### Mouse model of invasive pulmonary aspergillosis.

Eight- to ten-week-old female BALB/c mice (Charles River, Senneville, Quebec, Canada) were neutrophil depleted by intraperitoneal injection of 200 μg of anti-Ly6G antibody (clone 1A8, BioXcell) every 48 h, beginning 1 day prior to infection. Mice were then infected intratracheally with 1 × 10^7^ A. fumigatus conidia in 50 μl of PBS plus 0.01% (vol/vol) Tween 80 (PBS-T) or with PBS-T alone for uninfected controls. Doxycycline (Sigma) was administered by intraperitoneal injection at doses ranging from 10 to 25 mg/kg by oral gavage (100 mg/kg doxycycline every 12 h) or by supplementation of drinking water (500 μg/ml doxycycline and 5% sucrose) and chow (625 mg/kg doxycycline; Envigo-Teklad) as indicated. Doxycycline-free control mice were given equal volumes of PBS. Serum doxycycline levels were determined by ultra-high-performance liquid chromatography coupled to mass-spectrometry (uHPLC-MS/MS) analyses at the Drug Discovery Platform of the Research Institute of the McGill University Health Centre (Montreal, Canada). For fungal burden studies, mice were euthanized 36 h postinfection, and their lungs were harvested and homogenized in PBS. The fungal burden was determined by quantification of pulmonary galactomannan (GM) content using a Platelia *Aspergillus* Ag kit (Bio-Rad) as previously described (55).

### Ethics statement.

All procedures involving mice were approved by the McGill University Animal Care Committee, under protocol number AUP-2015-7674, and followed the University Animal Care Committee (UACC) guidelines as established by the Canadian Council on Animal Care (CCAC).

### RNA sequencing analysis.

A total of 1 × 10^6^ conidia/ml were grown in AMM for 10 h at 37°C and then treated with 20 μg/ml doxycycline or dH_2_O for 30 min. The resulting fungal biomass was crushed under liquid nitrogen before extracting RNA using an RNeasy minikit (Qiagen) according to the manufacturer’s instructions. cDNA libraries were prepared with a NEBNext Ultra II RNA Library Prep kit (NEB) and sequenced with an Illumina HiSeq4000 (Illumina) at the McGill University and Genome Quebec Innovation Centre (Montreal, Canada). Sequencing yielded a median of 43.7 million paired-end reads with at least 25 million paired-end reads per library. The quality of the reads was assessed with FastQC and MultiQC (56). Sequenced reads were aligned to the A. fumigatus Af293 (ASM265v1) genome and transcriptome using STAR (57). The transcript level quantification was performed with Salmon quant adjusting for both sequencing and GC bias (58). The quantification matrix was imported to R environment using lengthScaledTPM from txtimport (59). Samples were normalized by library depth and low-expression transcripts (fewer than 50 reads in two replicates of a condition) were removed. Normalized counts were transformed to log_2_ counts per million (cpm) reads using voom (60). Differential gene expression analysis was performed with limma framework (61). To compare the transcriptome effect of *brlA* induction in A. fumigatus a linear model with a nested design was used. The impact of doxycycline in the inducible *brlA* overexpression strain and the wild-type parental strain of A. fumigatus was detected by independent coefficients, which were contrasted to identify doxycycline-independent transcriptome changes. To assess transcriptome changes at baseline, the inducible *brlA* overexpression mutant and the wild-type parental strain of A. fumigatus were compared without doxycycline treatment. The volcano and PCA plots were produced with ggplot2 and heatmaps with heatmap.2 R packages (62). The FDR *P* values were obtained by adjusting the raw *P* values using the Benjamini-Hochberg method in limma (61).

### Quantification of hyphal cell wall-bound galactosaminogalactan.

A total of 1 × 10^5^ conidia were inoculated per well in 24-well plates containing sterile coverslips and cultured for 8 h in liquid RPMI 1640 media (Wisent) containing doxycycline at the indicated concentrations. Coverslips were washed with PBS, stained with 50 μg/ml of fluorescein-tagged soybean agglutinin lectin (SBA-FITC), fixed in 4% paraformaldehyde, mounted, and imaged as described above, with an excitation of 495 nm and an emission of 519 nm. A series of images in the z-plane were obtained to encompass whole hyphae and converted into maximum intensity projections using ZEN black edition software (Zeiss). The MFI of the stained hyphae was quantified by measuring the mean pixel density using ImageJ software (60).

### Trehalose quantification.

A total of 2 × 10^6^ conidia/ml were incubated in AMM with 0.08 μg/ml doxycycline or dH_2_O for 12 h at 37°C with agitation. The biomass was collected, lyophilized, and processed to extract soluble trehalose as previously described (63). Briefly, 100-μl portions of cell extracts were combined with equal volumes of 0.2 M sodium citrate at pH 5.5 and incubated with 3 mU of porcine kidney trehalase (Sigma) or an equal volume of trehalase buffer for 18 h at 37°C. The total glucose in the samples was then quantified by a glucose oxidase (GO) assay (Sigma). Samples were normalized to trehalase-untreated samples to control for the presence of glucose prior to trehalase treatment.

### Data availability.

The RNA library has been made available at the GEO repository, under accession number GSE143601.
